# Efficacy of a novel drill dilator in overcoming severe biliary strictures with balloon enteroscopy

**DOI:** 10.1055/a-2271-4215

**Published:** 2024-03-08

**Authors:** Haruka Toyonaga, Tsuyoshi Hayashi, Masayo Motoya, Toshifumi Kin, Kuniyuki Takahashi, Akio Katanuma

**Affiliations:** 1 Center for Gastroenterology, Teine Keijinkai Hospital, Sapporo, Japan

In patients with altered anatomy, biliary strictures are widely overcome using balloon enteroscopy-assisted endoscopic retrograde cholangiopancreatography (BE-ERCP). As the balloon enteroscope has a small-diameter channel and scope maneuverability is poor, severe biliary strictures are often difficult to overcome. In such cases, dilation has previously been achieved using screw-type stent retrievers 1 2 . Recently, however, a novel drill dilator (Tornus ES; Asahi Intecc Co., Ltd., Aichi, Japan) has been developed specifically for penetrating and dilating strictures 3 4 5 .

**Case 1:** A woman aged in her 60s, with liver metastasis after subtotal stomach-preserving pancreaticoduodenectomy (SSPPD) for pancreatic head cancer presented with cholangitis due to a severe malignant stricture in the left hepatic bile duct ( Fig. 1 ). Attempts to place a plastic stent using a balloon enteroscope (SIF-H290S, Olympus Co., Tokyo, Japan) were challenging because of the extreme hardness of the stricture. Despite trying another dilator, only a catheter was able to pass through. The novel drill dilator easily traversed and dilated the stricture, allowing for successful stent placement ( Fig. 2 , Video 1 ).

**Fig. 1:**
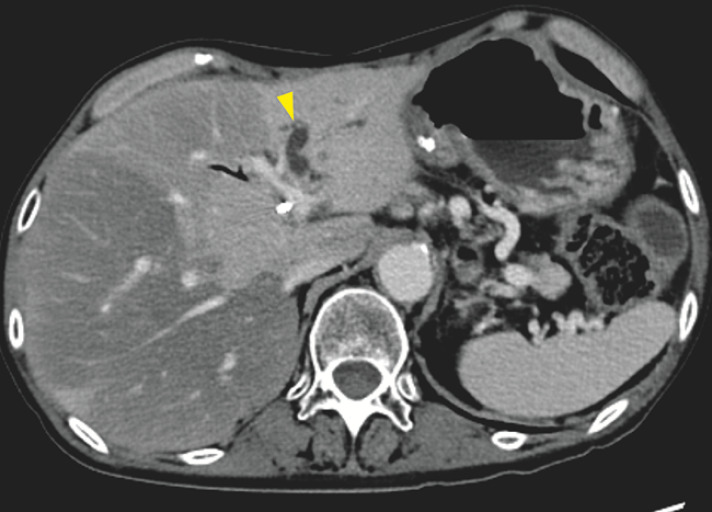
Contrast-enhanced computed tomography image of the dilated left hepatic bile duct (yellow arrowhead), which was obstructed by metastasis of pancreatic cancer on the biliary hilum.

**Fig. 2:**
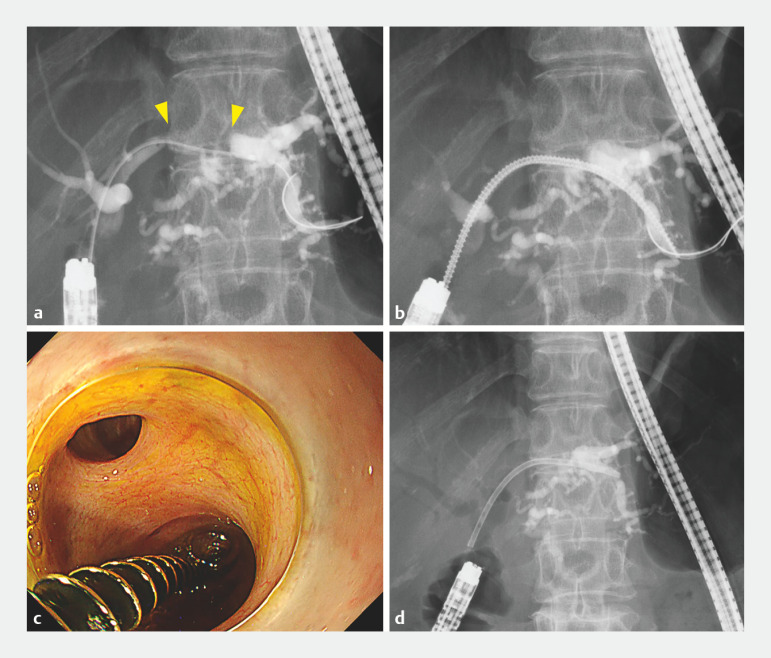
Balloon enteroscopy-assisted endoscopic retrograde cholangiopancreatography was performed to place a plastic stent in the occluded bile duct. **a** The left hepatic duct was rigidly obstructed for a long distance (yellow arrowheads), and only a catheter was able to pass through. **b** The novel drill dilator could easily break through and dilate the stricture by simply rotating, without strong pushing. **c** Endoscopic image of the drill dilator. **d** After dilation, a 7 Fr plastic stent was successfully deployed in the left hepatic duct.

A novel drill dilator showed efficacy in overcoming severe biliary strictures during balloon enteroscopy-assisted endoscopic retrograde cholangiopancreatography in patients with altered anatomy, even when scope maneuverability and device options were limited.Video 1

**Case 2:** A man aged in his 70s experienced cholangitis with intrahepatic biliary obstruction due to recurrence of distal bile duct cancer after SSPPD. It was determined that the obstruction in the posterior branch, where stenting had not been successful, was the main cause of cholangitis. The stricture was very tight and significantly angulated, making it impossible to traverse with any devices under BE-ERCP ( Fig. 3 ). The rotational force and adequate flexibility of the novel drill dilator enabled the stricture to be successfully traversed and dilated, followed by successful stent placement ( Fig. 4 , Video 1 ).

In BE-ERCP, where the range of usable devices and maneuverability are limited, the novel drill dilator proved to be useful in dilating severe biliary strictures, even in patients with altered anatomy.

**Fig. 3:**
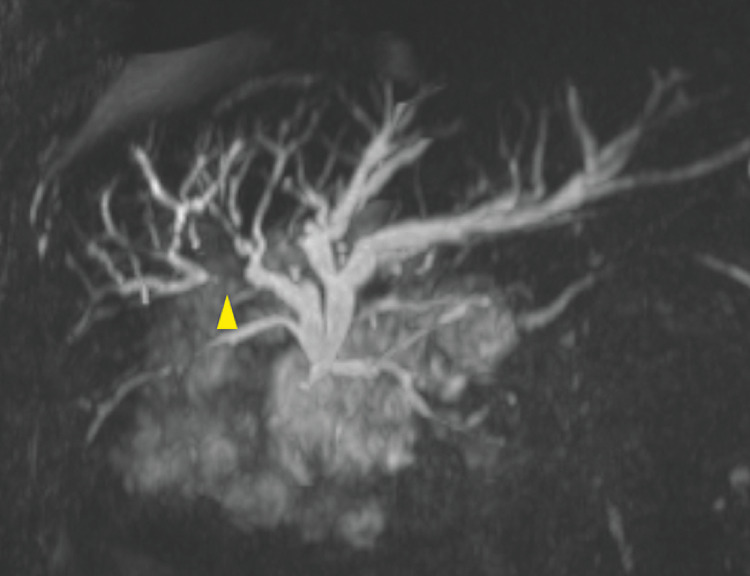
Magnetic resonance cholangiopancreatography image. The right posterior branch was occluded by the recurrence of biliary cancer (yellow arrowhead).

**Fig. 4:**
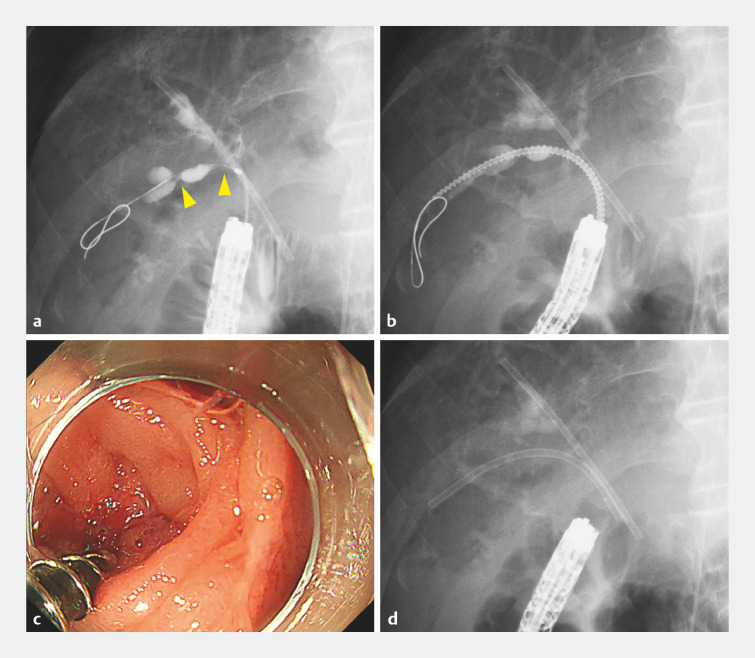
Use of the novel drill dilator for severe biliary structure. **a** The posterior branch of the right bile duct was rigidly stenosed in several places (yellow arrowheads) and at an acute angle to the enteroscope, making it impassable by any device. **b** The novel drill dilator could easily break through the stenosis without any resistance, despite the poor conditions. **c** Endoscopic image of the malignant stenosis being dilated by the drill dilator. **d** Fluoroscopic image after successful stent deployment.

Endoscopy_UCTN_Code_TTT_1AR_2AG

## Notes

Endoscopy E-Videos https://eref.thieme.de/e-videos
*E-Videos* is an open access online section of the journal *Endoscopy* , reporting on interesting cases and new techniques in gastroenterological endoscopy. All papers include a high-quality video and are published with a Creative Commons CC-BY license. Endoscopy E-Videos qualify for HINARI discounts and waivers and eligibility is automatically checked during the submission process. We grant 100% waivers to articles whose corresponding authors are based in Group A countries and 50% waivers to those who are based in Group B countries as classified by Research4Life (see: https://www.research4life.org/access/eligibility/ ). This section has its own submission website at https://mc.manuscriptcentral.com/e-videos .
